# Dataset for deformation behavior of pure titanium grade 2 materials during continuous extrusion

**DOI:** 10.1016/j.dib.2022.108309

**Published:** 2022-05-21

**Authors:** Mulualem Hailu Besha, Devendra Kumar Sinha, Getenet Asrat, Dawit Gudeta

**Affiliations:** a Mechanical Engineering Department, Manufacturing Engineering, School of Mechanical and Industrial Engineering, Dire Dawa University, Dire Dawa 1362, Ethiopia; b Mechanical Engineering Department, School of Mechanical, Chemical and Materials Engineering, Adama Science and Technology University, Adama and 1888, Ethiopia; c Materials Science & Engineering, School of Mechanical, Chemical and Materials Engineering, Adama Science and Technology University, Adama and 1888, Ethiopia

**Keywords:** Continuous extrusion, CP- Titanium grade 2, Feedstock temperature, DEFORM-3D, Extrusion wheel velocity, FEM

## Abstract

There is a huge application and demand for titanium alloys with excellent upgraded mechanical, metallurgical, and material properties in modern industries. To fulfill the demand of modern industries metal forming process is highly desirable. Among all metal forming processes, a special type of cold forming called the continuous extrusion process has been highly appropriate to fulfill the demands. The theoretical analysis has been carried out through Upper Bound Method. The numerical simulation has been carried out through the three-dimensional finite element tool DEFORM-3D. The experimental plan and design have been carried out using Taguchi (2^3) array methods on the MINITAB platform by considering extrusion wheel velocity and feedstock temperature as chief extrusion parameters. The experimental validation process was executed on 12.5 mm CP- Titanium grade 2 feedstock materials using a TBJ350 CONFORM machine setup. The optimization process of parameters for the optimum value of the response variable was carried out through Grey Relational Analysis.

## Specification Table

**Table:** 

Subject	Mechanical Engineering
Specific subject area	Metal forming
Types of data	Table, figure, equations, and text file
How data were acquired	Theoretical analysis has been analyzed using upper bound technology, Numerical simulation has been run through the Deform-3D platform, and Experimental validation has been done using the TBJ350 CONFORM machine setup.
Data format	Raw and Analyzed
Parameters for data collection	The Continuous Extrusion Process parameters are Extrusion wheel velocity 4, 8, and 12 RPM, and Feedstock Preheating temperatures 200, 400, and 600 °C.
Description for data collection	Deformation behavior of CP-Titanium grade 2 feedstock materials through the Continuous Forming process has been carried out. The theoretical analysis, numerical simulation, and experimental validation have been carried out. The theoretical analysis has been carried out through Upper Bound Method. The numerical simulation has been carried out through the three-dimensional finite element tool DEFORM-3D. The experimental plan and design have been carried out using Taguchi (2^3) array methods in the MINITAB platform by considering extrusion wheel velocity and feedstock temperature as chief extrusion parameters. The experimental analysis validation was executed on 12.5 mm CP- Titanium grade 2 feedstock materials using a TBJ350 CONFORM machine setup. Finally, the optimization process of parameters for the optimum value of the response variable was carried out through Grey Relational Analysis.
Data source location	Adama Science and Technology University, Adama, Ethiopia.Banira Industrial Estate, Jungalpur, Howrah, India.
Data accessibility	Dataset is provided in this article, and The relevant raw and analyzed data can be found in the mentioned repository, DOI, and URL.Mendeley Data, DOI: 10.17632/4rw48pzvgd.1https://doi.org/10.17632/4RW48PZVGD.1

## Value of the Data

• The dataset helps to realize the optimum parameter conditions of CP-Ti-Grade 2 deformation through the Continuous Extrusion process and achieve better mechanical and metallurgical properties.
• The dataset reveals the optimum parameter interaction during the Continuous Extrusion process, which is highly helpful for metal forming researchers and industries in the Continuous Forming process of non-ferrous alloys, especially on titanium feedstock materials.
• The dataset presented is analyzed through theoretical analysis, numerical simulation, and experimental validation and optimized using the GRA technique, which can be applied for further analysis using other multivariate optimization techniques like the DEAR approach, Genetic algorithm, and Factor analysis.

## 1 Data Description

In this article, both raw and analyzed datasets are accessible, correlated with deformation analysis of Commercially Pure Titanium Grade 2 material through a continuous extrusion process. Table 1, 2, 3, and 4 illustrates the elemental composition, physical, mechanical, and thermal properties of CP-Titanium grade 2 feedstock material. The data presented in this table is provided by the suppliers CP-Titanium grade 2 feedstock material [1]. The experimental plan and design of the chief parameters of the process have been elucidated using Taguchi L9 (2^3) orthogonal array, as shown in Table 5. The theoretical analysis results of CP-Titanium Grade 2 Feedstock material using the upper bound method have been tabulated in Table 5. Table 6 illustrates the Numerical simulation results of CP- Titanium grade 2 material through DEFORM-3D. Table 7 depicts the Simulation Results for CP titanium grade 2 feedstock material. Finally, Table 8 shows the Experimental validation Results for CP titanium grade 2 feedstock material. The rationale behind the theoretical analysis is to determine and understand the deformation behavior of cp-titanium grade 2 material during continuous forming processes under various ideal assumptions, such as the extrusion wheel (driving wheel) as a rigid and non-friction side plate. The rationale behind running numerical simulations is to determine and understand the deformation behavior of cp-titanium grade 2 material during continuous forming processes through the finite element method through which the results can be approximated to a great extent, including various assumptions such as workpiece material is assumed to be rigid visco-plastic, the material is homogeneous and isotropic, there is no strain hardening, interfaces are either frictionless, or sticking friction prevails.

**Table 1:** Elemental composition of CP-Titanium grade 2 feedstock material [1].

Content	Nitrogen	Carbon	Hydrogen	Iron	Oxygen	Titanium
in wt.%	0.03	0.08	0.015	0.30	0.25	99.325

**Table 2:** Physical properties of CP-Titanium grade 2 feedstock material.

Physical properties	Metric
Density	4.51 g/cc

**Table 3:** Mechanical properties of CP-Titanium grade 2 feedstock material.

Mechanical	Hardness,	Tensile strength,	Tensile strength,	Elongation	Modulus of	Poisons	Shear
properties	Vickers	ultimate	yield	at break	elasticity	ratio	modulus
Metric	145	344 MPa	275-410 MPa	20 %	105 GPa	0.37	45 GPa

**Table 4:** Thermal properties of CP-Titanium grade 2 feedstock material.

Thermal properties	Specific heat capacity	Thermal conductivity	Melting point	Heat of fusion
Metric	0.523 J/g-°C	16.4 W/m-K	Max 1665 °C	325 J/g

**Table 5:** Theoretical Analysis results of CP-Titanium grade 2 Material Through Upper Bound Theorem.

Run	Extrusion wheel	Feedstock	Total required	Load	Power
Order	Velocity (RPM)	Temperature (°C)	pressure (MPa)	required (KN)	required (KW)
1	4	200	1662.78	20.78	108.83
2	4	400	732.49	9.16	47.96
3	4	600	372.55	4.66	24.38
4	8	200	1662.78	20.78	217.61
5	8	400	732.49	9.16	95.9
6	8	600	372.55	4.66	48.88
7	12	200	1662.78	20.78	326.4
8	12	400	732.49	9.16	143.88
9	12	600	372.55	4.66	73.28

**Table 6:** Numerical simulation results of CP- Titanium Grade 2 material through DEFORM-3D.

Run Order	Extrusion wheel Velocity(RPM)	Feedstock Temperature(°C)	Effective stress(MPa)	Maximum temperature(°C)	Total load(kN)	Torque(kN-m)	EffectiveStrain(mm/mm)	Damage value	Power required(KW)
1	4	200	1020	864	5.81	21.4	20.3	3.05	117.86
2	4	400	746	746	3.93	15.6	7.97	3.05	52.56
3	4	600	575	597	2.90	13	7.97	2.41	25.31
4	8	200	981	921	5.81	21.9	12.9	2.82	230.88
5	8	400	763	966	3.31	13.7	23.8	3.02	99.7
6	8	600	479	833	3.28	11.3	5.05	2.74	52.7
7	12	200	935	928	6.84	23	26.3	3.21	339.95
8	12	400	647	931	3.53	12	17.5	3.07	155.39
9	12	600	485	914	4.98	13.9	7.44	2.68	78.23

**Table 7:** Simulation Results of deformation of CP titanium grade 2 feedstock material.

Sample		Preheating	Extruded rod	Extrusion		Total
No.	Shape	temperature (°C)	diameter (mm)	Ratio	RPM	Power (kW)
1	Circular Rod	1000	6	4.34	4	3.48
2			8	2.44	4	3.71
3			10	1.56	4	4.05

**Table 8:** Experimental validation Results of deformation of CP- Titanium Grade 2 feedstock material.

Sample		Preheating	Extruded rod	Extrusion		Total
No.	Shape	Temperature (°C)	diameter (mm)	Ratio	RPM	Power (kW)
1	Circular Rod	1000	6	4.34	4	4.22
2			8	2.44	4	3.81
3			10	1.56	4	3.62

All in all, the main reasons behind running both methods are to identify and understand the optimum conditions and ranges of each input parameter for the deformation processes of cp-titanium grade 2 material through continuous forming within fewer power consumptions, safe working conditions, especially for extrusion wheel, shoe, and abutment materials and to estimate the differences in results occurring due to these approaches.

The investigation on analysis of a robust new design for titanium alloy prick hole extrusion was carried out through FEA and Taguchi methods. The effect of die semi angle, die hole diameter, friction factor, ram velocity, and temperature of billet were investigated on the strain, stress, and damage value. The optimum value of input parameters was investigated for achieving the perfect extrusion [7]. The multi-response optimization of hot extrusion process parameters using FEA and Grey Relational based Taguchi method was carried out on hot direct extrusion of AA6061alloy. The numerical simulation was carried out through DEFORM-3D. The statistical significance of the process parameters was carried out through Analysis of Variance (ANOVA). The degree of significance of die angle was found to be highest followed by ram speed and coefficient of friction [8]. The investigation on sensitivity analysis of die structural and process parameters in porthole die extrusion of magnesium alloy tube was carried out using the Taguchi method. The simulation of the welding process of metal streams for calculation of peak extrusion load and the temperature was carried out through Lagrangian- Eulerian algorithm. The analysis of welding pressure, temperature, effective strain rate, and stresses on the welding plane were investigated through the J-welding criterion [9].

The numerical simulation of 12.5 mm diameter Titanium alloy feedstock material has been carried out using the Finite Element simulation tool DEFORM-3D.

The basic equations of the rigid-plastic finite element are as follows [10]:

Equilibrium equation:

(1) $$\sigma_{ij,j}=0$$

Compatibility and incompressibility equations

(2) $$\begin{array}{c} {\dot{\varepsilon }}_{ij}=\frac{1}{2}\left(u_{ij}+u_{ji}\right)\\ {\dot{\varepsilon }}_{v}=u_{ij}=0 \end{array}$$

Constitutive equations:

(3) $$\sigma^{\prime }{}_{ij}=\frac{2\bar{\sigma }}{2\dot{\bar{\varepsilon }}}{\dot{\varepsilon }}_{ij},\;\;\bar{\sigma }=\sqrt{\frac{3}{2}{\sigma^{\prime }}_{ij}{\sigma^{\prime }}_{ij}\;},\;\;\;\dot{\bar{\varepsilon }}=\sqrt{\frac{3}{2}\left({\dot{\varepsilon }}_{ij}{\dot{\varepsilon }}_{ij}\right)\;\;\;}$$

Boundary conditions:

(4) $$\sigma_{ij}n_{i}=F_{j}\;On\;\;S_{F},u_{i}=U_{i}\;\;on\;\;S_{U}$$

where $\sigma_{ij}$ and ${\dot{\varepsilon }}_{ij}$ are the stress and the strain rate, respectively, $\bar{\sigma }$ and $\dot{\bar{\varepsilon }}$ are the effective stress and the effective strain rate, respectively$.\;F_{j}$ is the force on the boundary surface of $S_{F}$, and $U_{i}$is the deformation velocity on the boundary surface of $S_{U}$. The weak form of rigid-plastic FEM can be determined by applying the variation method to Eqs. (1) – (4), i.e.

(5) $$\int_{V}\bar{\sigma }\delta \bar{\varepsilon }dv\;+K\;\;\int \varepsilon_{V}\delta \varepsilon_{V}dV-\int_{S_{F}}F_{i}\delta u_{i}dS=0$$

Where *V* and *S* are the volume and the surface area of the material, respectively, and *K* is the penalty constant.

Yielding criteria which are used for solving the problem is Von Mises (in the Deform3D). Eqs. (6) And (7) show effective strain and effective stress respectively.

(6) $$\bar{\varepsilon }=\frac{\sqrt{2}}{3}\sqrt{{\left(\varepsilon_{1-}\varepsilon_{2}\right)}^{2}+\;{\left(\varepsilon_{2-}\varepsilon_{3}\right)}^{2}+{\left(\varepsilon_{3-}\varepsilon_{1}\right)}^{2}}$$

(7) $$\bar{\sigma }=\frac{1}{\sqrt{2}}\sqrt{{\left(\sigma_{1-}\sigma_{2}\right)}^{2}+\;{\left(\sigma_{2-}\sigma_{3}\right)}^{2}+{\left(\sigma_{3-}\sigma_{1}\right)}^{2}}$$

Where $\varepsilon_{i}$ and $\sigma_{i}$ are principle strain and principal stress in the direction i respectively.

The upper bound theorem [2] states that among all possible kinematically admissible velocity fields, the one that minimizes the total power φ*_T_* is the actual velocity field.

(8) $$\phi_{T}=\int_{\Omega }{\sigma^{\prime }}_{ij}{}^{*}{\dot{\in }}_{ij}^{*}d\Omega +\;\int_{S_{i}}\tau {\left|\Delta v_{i}\right|}_{S_{i}}^{*}dS_{i}$$

The first term expresses the internal power of deformation over the volume of the deformation zone. In contrast, the second term represents the power dissipated in shearing the material over the velocity discontinuity surfaces and at the tool-work interface (i.e., frictional power). Here asterisk (*) indicates that the values of stress, strain rate, and velocity discontinuity are obtained from an assumed kinematically admissible velocity field.

Table 8 portrays the Experimental validation Results of CP titanium grade 2 feedstock material deformation. Table 9 illustrates the validation of power consumption for deformation of CP-Titanium grade 2 material. Finally, Table 10 represents the Test result of ANOVA for effective stresses of CP- Titanium Grade 2 feedstock material. So as tabulated in Table 10, ANOVA determines the significance effect and contribution of each (Extrusion wheel velocity, Feedstock Temperature) parameter on the effective stresses of feedstock material during the continuous forming processes to get optimum (minimum) required deformation load and power consumption.

**Table 9:** Validation of power consumption for deformation of CP-Titanium grade 2 material.

Parameters		Simulation PowerResult (kW)	Experimental PowerResult (kW)	% Error (Between Simulation & Experiment)
Feedstock Diameter =12.5mm,Preheating Temperature = 1000 °CExtrusion Wheel Velocity = 4 RPM	Extrusion ratio=4.34	4.05	4.22	4.3
	Extrusion ratio=2.44	3.71	3.81	2.4
	Extrusion ratio=1.56	3.48	3.62	3.9

**Table 10:** Test result of ANOVA for effective-stresses of CP- Titanium grade 2 feedstock material.

Source	DF	Seq SS	Adj MS	F-Value	P-Value	Contribution
Model	5	335632	67126	99.81	0.002	99.40%
Linear	2	332959	166479	247.53	0.000	98.61%
Extrusion wheel velocity (RPM)	1	12331	12331	18.33	0.023	3.65%
Feedstock Temperature (C)	1	320628	320628	476.73	0.000	94.96%
Square	2	2592	1296	1.93	0.290	0.77%
Extrusion wheel velocity (RPM)*Extrusion wheel velocity (RPM)	1	968	968	1.44	0.316	0.29%
Feedstock Temperature (C)*Feedstock Temperature (C)	1	1624	1624	2.42	0.218	0.48%
2-Way Interaction	1	81	81	0.12	0.751	0.02%
Extrusion wheel velocity (RPM)*Feedstock Temperature (C)	1	81	81	0.12	0.751	0.02%
Error	3	2018	673			0.60%
Total	8	337650				100.00%

DF: degree of freedom; Adj SS: adjusted sums of squares; Adj MS: adjusted mean

**Fig. 1:**
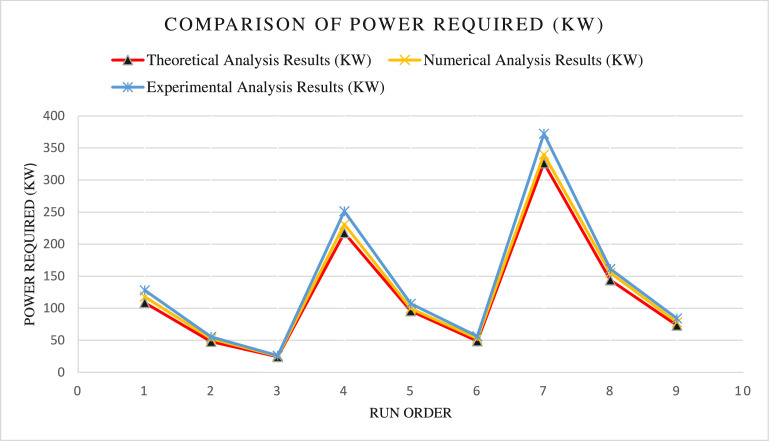
Comparison of power required for deformation process

The Theoretical analysis, Numerical Simulation, and Experimental analysis of power consumption results for deformation of CP- Titanium Grade 2 material have been illustrated in Table 11 and Fig. 1 above and are in good agreement, thereby maintaining the error up to 5 %.

**Table 11:** Comparison of Theoretical, Numerical, and Experimental Analysis of Required Power (KW) for deformation of CP- Titanium Grade 2 feedstock material.

Run Order	Extrusion wheel Velocity (RPM)	Feedstock Temperature (°C)	Theoretical Analysis Results (KW)	Numerical Analysis Results (KW)	Experimental Analysis Results (KW)
1	4	200	108.83	117.86	127.99
2	4	400	47.96	52.56	55.50
3	4	600	24.38	25.31	26.18
4	8	200	217.61	230.88	251.28
5	8	400	95.9	99.7	107.26
6	8	600	48.88	52.7	56.47
7	12	200	326.4	339.95	372.25
8	12	400	143.88	155.39	161.45
9	12	600	73.28	78.23	84.16

## 2 Experimental Design, Material and Methods

### 2.1 Materials

**Fig. 2:**
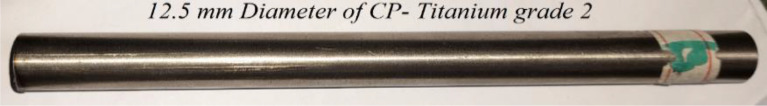
12.5 mm diameter of Commercially Pure titanium grade 2 feedstock material.

Commercially Pure titanium grade 2 circular rod material size of 12.5 mm diameter was utilized as feedstock material which is deformed through a continuous extrusion process whose elemental composition and properties are depicted in Tables 1, 2, 3 and 4. Fig. 2 depicts the physical appearance of CP-Ti grade 2 feedstock material before the deformation process.

**Fig. 3:**
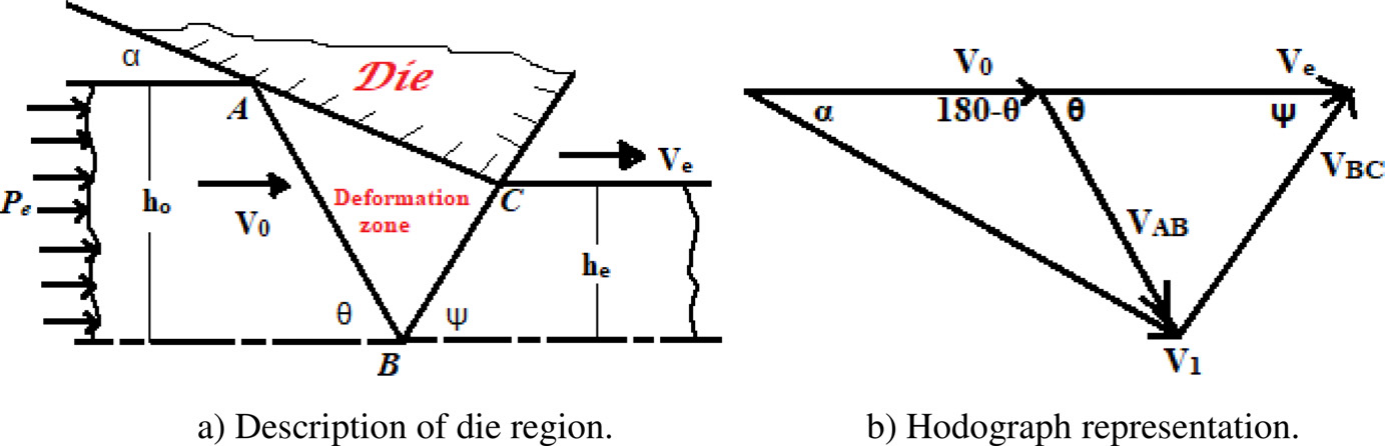
a) Description of die region. b) Hodograph representation.

### 2.2 Methods

Theoretical analysis, Numerical simulation and experimental validation were implemented to identify the optimum process parameter conditions of deformation of CP-Titanium grade 2 feedstock material through the Continuous Extrusion Process. [3] The theoretical analysis was analyzed by similarity equations using the upper bound technique as shown in eqns. 1-5. The commercial finite element method code was applied through the DEFORM-3D platform [4] to conduct a numerical simulation analysis of deformation behavior by considering the input numerical data values as tabulated in Table 13. Numerical simulation analysis was evaluated by using the DEFORM-3D platform. The experimental validation process has been examined through commercial continuous extrusion setup TBJ350 machine having specification as shown in Table 12. Extrusion wheel velocity, feedstock temperature and extrusion ratio are the main parameters in the overall Continuous Extrusion processes of CP-Titanium grade 2 feedstock material.

**Table 12:** Experimental plan and Design table of Taguchi L9 (2^3) array.

Run Order	Extrusion wheel Velocity (RPM)	Feedstock Temperature (°C)
1	4	200
2	4	400
3	4	600
4	8	200
5	8	400
6	8	600
7	12	200
8	12	400
9	12	600

#### 2.2.1 In the die region

Here, According to the depreciation of the total rate of energy consumption *(*$\frac{dw_{T}\;}{dt}$*),* the value of θ_min_ = 90 and a corresponding value of die angle, α = 30. . There are two planes of tangential velocity discontinuity in the die region: AB and BC; while it crosses plane AB, the tangential velocity discontinuity is V_AB_. Similarly, while it crosses plane BC, the tangential velocity discontinuity is V_BC,_ as shown on the hodograph representation.

Average extrusion pressure in the die region $\left(p_{e_{d}\;}\right)$ can be calculated as,

(9) $$p_{e_{d}\;}=\;\frac{k\sin\alpha }{h_{0}\text{sin}\left(\theta -\alpha \right)}\;\left[\;\frac{h_{0}}{sin\theta }+\;\frac{h_{e}sin\theta }{sin^{2}\varphi }\right]\;$$

#### 2.2.2 In the groove region

Average extrusion pressure in the groove region ($p_{e_{G}\;})$ can be calculated as

(10) $$p_{e_{G}\;}=\;\frac{k}{H}\;\left[\;m_{2}\left(L+\;h_{0}\right)+m_{1}L+m_{3}\frac{H^{2}}{h_{0}}\;+\;\frac{h_{0}^{2}+H^{2}\;}{h_{0}}\right]$$

Where $m_{1}\;is$ the friction coefficient on the shoe, $m_{2}$ is the friction coefficient inside the groove, $m_{3}$ is the friction coefficient on the abutment, *k* is shear stress of the feedstock material, *H* is the width of feedstock material, *h_0_* die chamber height and *L* is the length of the container (die). Then, the total extrusion pressure ($p_{e_{T}\;})\;$is the sum of average extrusion pressure in the die and average extrusion pressure in the groove,

(11) $$p_{e_{T}\;}=\;p_{e_{d}\;}+p_{e_{G}\;}\;$$

The force required to carry out the extrusion process can be calculated as,

(12) $$F\;=\;p_{e_{T}\;}*H\;\left(N\right)$$

Then, the power required during the continuous forming process can be calculated as,

(13) $$P\;=\;\frac{\;F*H*2\pi *N}{60}\;\left(W\right)$$

Where *F*- the force required for deformation, *H*- the width of material, mm and *N*- Extrusion wheel velocity, rpm.

**Fig. 4:**
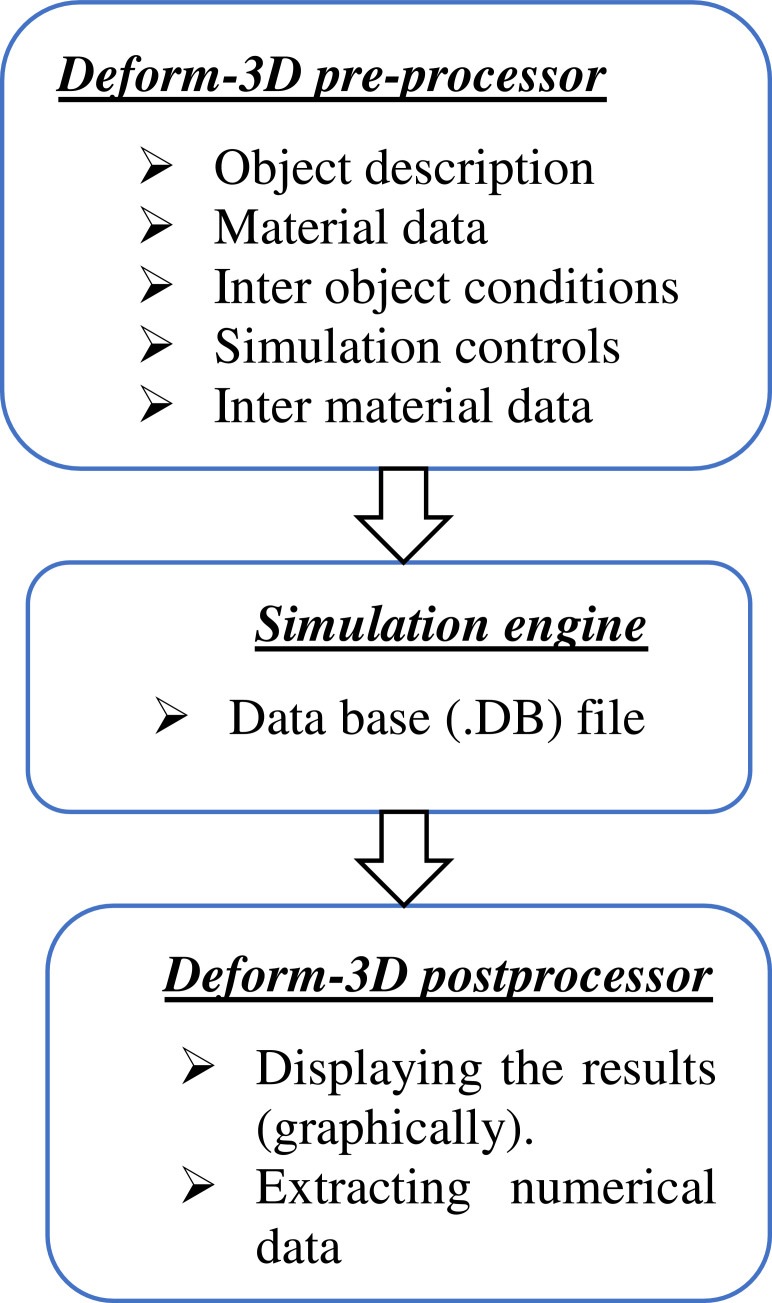
The flow processes of the Deform-3D tool system [5].

As illustrated in Table 12 below in the Experimental plan and Design table of the Taguchi L9 (2^3) orthogonal array two variables are considered as a factor with three levels. Table 13 depicts the numerical simulation data of CP-Ti grade 2 that include geometry data, simulation data and material data. The overall flow processes of the DEFORM-3D tool system have been illustrated in Fig. 4. Table 14 illustrates details of tetrahedron mesh elements.

**Table 13:** Numerical Simulation data of CP -Titanium Grade 2.

***Geometry data***	
Wheel diameter, *D* (mm)	350
Die diameter, *d,* (mm)	6
Material width, *H* (mm)	9
Die length, *L(*mm)	10
***Simulation data***	
Wheel velocity,(RPM)	4/8/12
Environment temperature(°C)	25
Time steps(s)	5
Feedstock Temperature(°C)	200/400/600
***Feedstock Material data***	
Yield stress(MPa)	375
Strain rate sensitivity	0.22
Heat capacity(N/mm^2^/°C)	526.3
Conductivity (N/s/°C)	6.7
Convective heat transfer coefficient(N/s/°C/mm)	0.02
Emissivity coefficient	0.3
Heat transfer Coefficient (N/s/°C/mm)	30
Friction coefficient (Interface)	0.3 (Feedstock and Wheel groove),0.3 (Abutment and Feedstock), and0.1 (Die and Feedstock)

**Table 14:** Details of tetrahedron mesh elements.

S. No.	Objects	Mesh-Elements
1	Work piece	72055
2	Extrusion Wheel	148537
3	Extrusion shoe	24885
4	Coining Wheel	65307
5	Abutment	76391
6	Die	45589

**Table 15:** Specification of Commercial Setup of TBJ350 machine.

S. No.	Parameters	Values	Applications
1	Bed Length	5 meters	Setup is applicable for the extrusion of non-ferrous round rods, square rods, tubes and bus bars with a wide range of extrusion ratios.
2	Weight	5 ton	
3	Motor Power	200 kW	
4	Extrusion Wheel Diameter	350 mm	
5	Feedstock Diameter	12.5 mm

**Table 16:** Experimental validation plans.

Exp. No	Preheating Temperature (°C)	Extrusion Wheel Velocity (RPM)	Extrusion Ratios (A^2^_0_/A^2^_f_)
1	1000	4	1.56
2			2.44
3			4.34

Specification and applications of Commercial Setup of TBJ350 machine have been tabulated in Table 15. Table 16 depicts the experimental validation plans of the process. For the case of experimental validation of power consumption, 1000 °C feedstock temperature and 4 rpm extrusion wheel velocity have been chosen as per the prediction made by theoretical and numerical simulation results of the power consumption. The theoretical and simulation results suggested that the power consumption decreases as the extrusion wheel velocity decreases and feedstock temperature increases. As a result, for experimental validation, the feedstock temperature of the work material has been selected in the form of the average values of hot working condition feedstock of temperature and lower extrusion wheel velocity to get the optimum value (lower) of machine power consumption.

**Table 17:** S/N ratios and Normalized S/N ratios.

			S/N ratios		Normalized S/N ratios	
Ex.	Effective	Power	Effective-	Power	Effective-	Power
no	stress (MPa)	consumption (KW)	Stress	Consumption	Stress	Consumption
1	1020	117.86	-60.17	-41.43	1.000	0.79
2	746	52.56	-57.46	-34.41	0.586	0.55
3	575	25.31	-55.19	-28.15	0.242	0.33
4	981	230.88	-59.83	-47.27	0.948	1.00
5	763	99.70	-57.65	-39.97	0.616	0.74
6	479	52.70	-53.61	-34.44	0.000	0.55
7	935	339.95	-59.42	-25.31	0.885	0.22
8	647	155.39	-56.22	-21.91	0.398	0.10
9	485	78.23	-53.72	-18.94	0.016	0.00

**Table 18:** Grey Relational coefficient and grey relational grade.

	Normalized S/N ratios		Quality loss		Grey Relational coefficient		
Ex.	Effective-	Power	Δ*_Effective-_*	Δ*_Power_*	*GC_Effective-_*	*GC_Power_*	Grey
no	Stress	Consumption	*_Stress_*	*_Consumption_*	*_Stress_*	*_Consumption_*	grade
1	1.000	0.79	0.000	0.21	1.000	0.826	0.913
2	0.586	0.55	0.414	0.45	0.707	0.687	0.697
3	0.242	0.33	0.758	0.67	0.569	0.598	0.584
4	0.948	1.00	0.052	0.00	0.951	1.000	**0.976**
5	0.616	0.74	0.384	0.26	0.723	0.794	0.759
6	0.000	0.55	1.000	0.45	0.500	0.688	0.594
7	0.885	0.22	0.115	0.78	0.897	0.560	0.729
8	0.398	0.10	0.602	0.90	0.624	0.530	0.577
9	0.016	0.00	0.984	1.0	0.504	0.500	0.502

**Table 19:** Mean of MRPI values and ranking of factors effect.

Factors		Extrusion Wheel Velocity (A)	Feedstock Temperature (B)
levels	1	0.7312	0.8723*
	2	0.776*	0.6775
	3	0.6025	0.5598
Rank		2	1

### 2.3 Experimental design

#### 2.3.1 Design of experiments

**Fig. 5:**
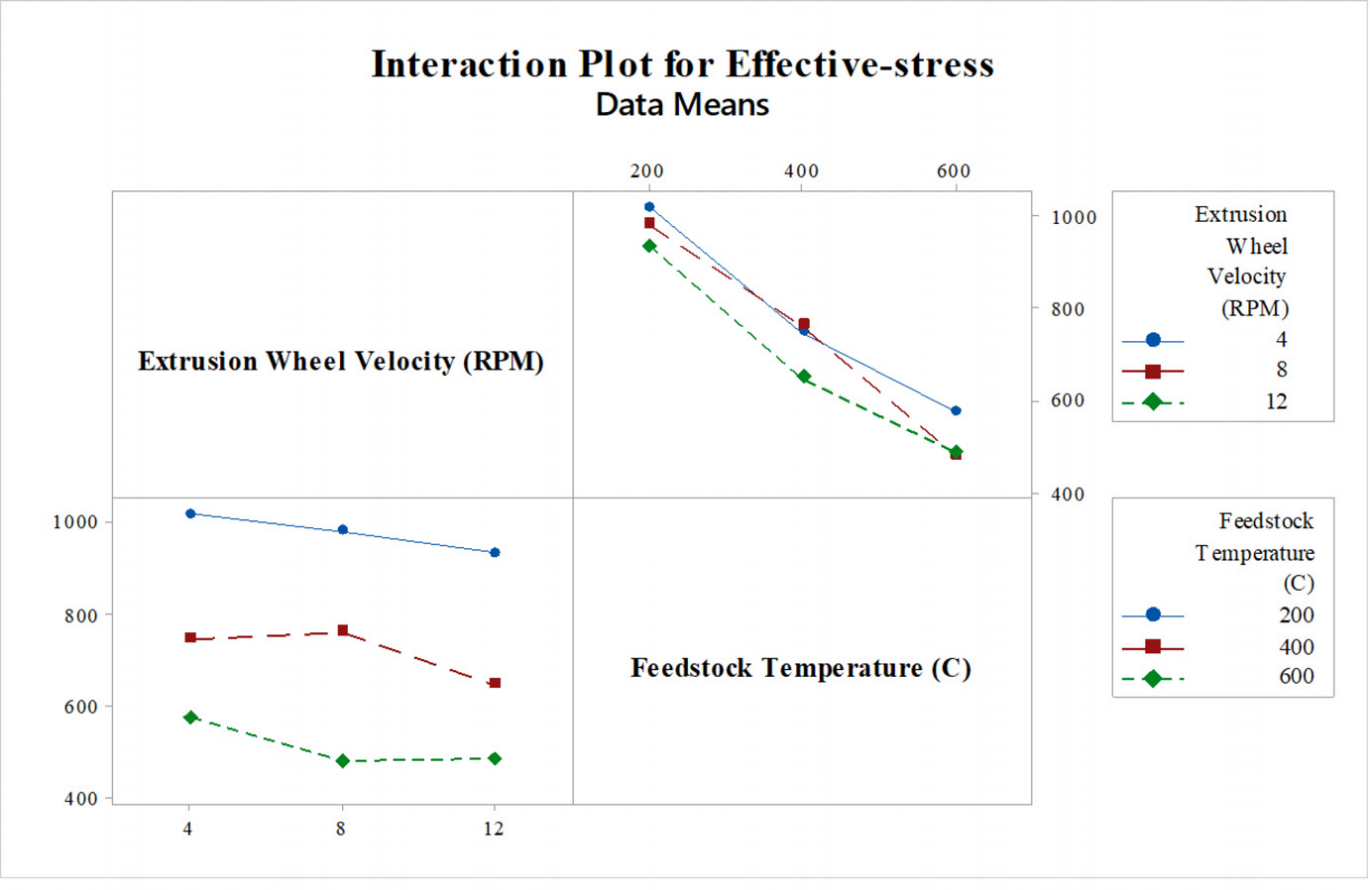
Commercial Continuous Extrusion setup [5]

**Fig. 6:**
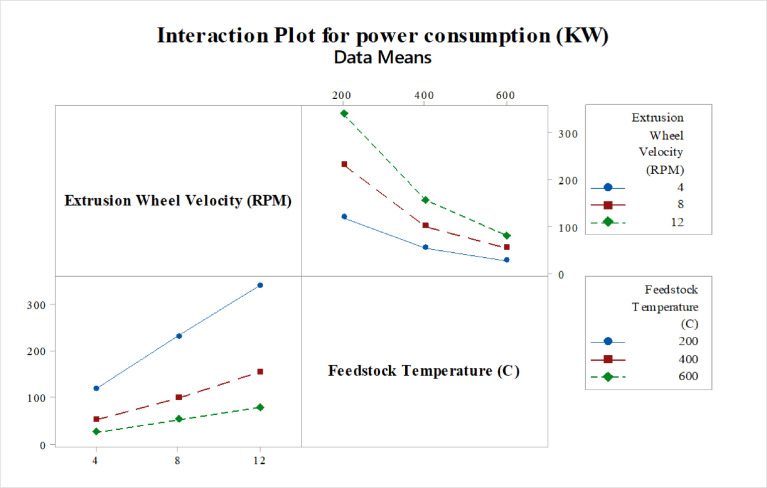
Interaction plot for Effective-stress

Design of experiment (DOE) is a method of defining and investigating all possible conditions involving multiple factors, parameters, and variables in the experimental analysis Taguchi method is a commonly used problem-solving tool that can improve the product's performance, process design, and system. And also, it's is a process optimization method that is based on 8-steps of planning, conducting, and evaluating the results of matrix experiments to determine the best levels of control factors. The primary goal is to keep the variance in the output very low, even in the presence of noise inputs. As shown below, Fig. 5. demonstrates the interaction plot of a mean of effective stress. In this interaction plot the lines are not parallel which indicates, the relationship between Extrusion wheel velocity and feedstock temperature on the value of mean effective stress has a significant effect which clarifies the interaction between feedstock temperature and extrusion wheel velocity during the process on the results of effective-stresses of the feedstock material to, optimize the load required for the deformation of feedstock material during continuous forming processes. Therefore, the interaction between extrusion wheel velocity at 8 rpm and 600 °C feedstock temperature is the optimum combination of parameters for the deformation of feedstock material during the processes. Fig. 6. illustrates the interaction plot of a mean of power consumption. It indicates the interaction between extrusion wheel velocity and feedstock temperature on of TBJ350 conform machine during the processes. . In this interaction plot, the lines are not parallel. So, the interaction effect indicates that the relationship between Extrusion wheel velocity and feedstock temperature significantly affects the mean power consumption value, as its displayed in Fig. 6. below, the combination of extrusion wheel velocity at 4 rpm and 600 °C feedstock temperature indicates the optimum power consumption of the machine during the deformation process.

**Fig. 7:**
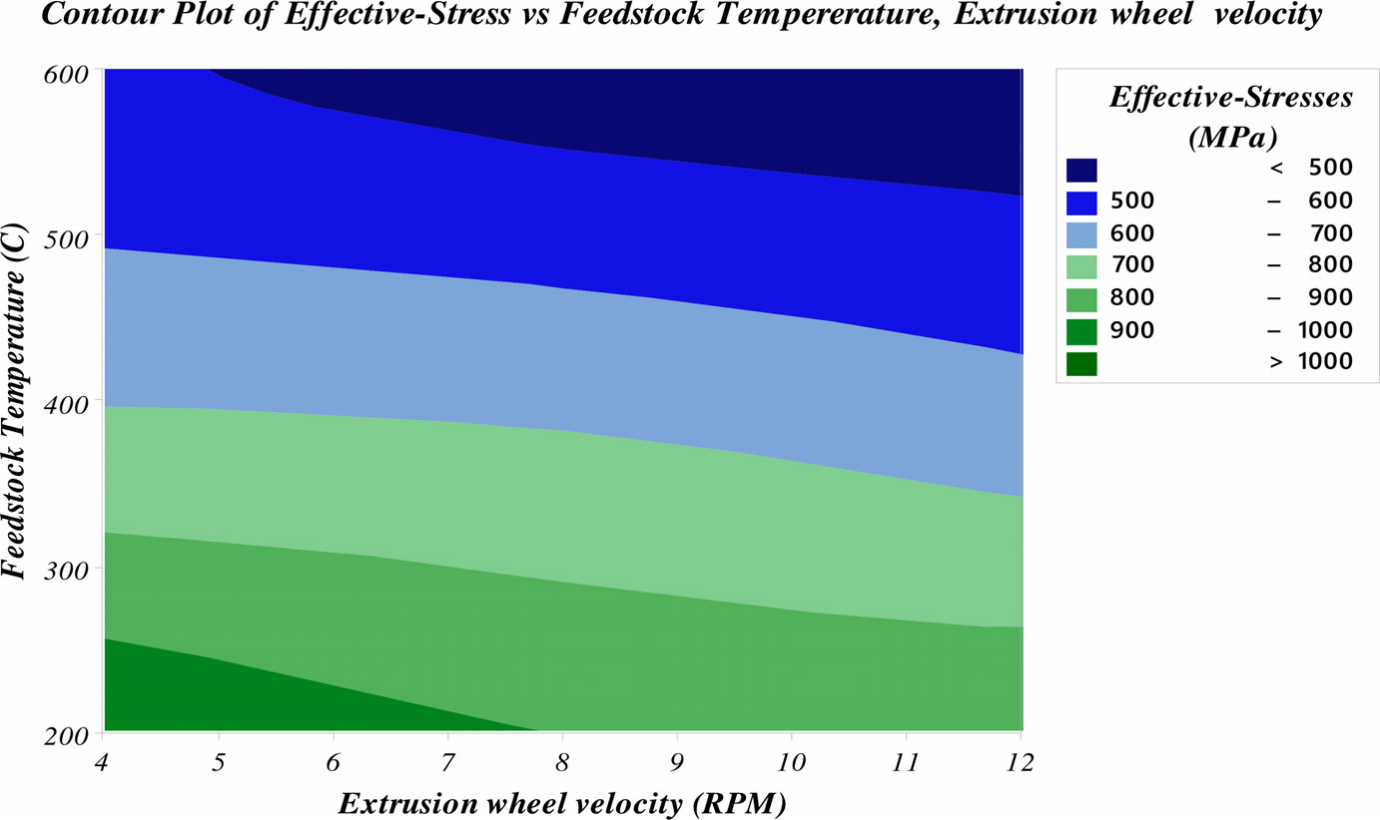
Interaction plot for power consumption

Fig. 7 shows a contour plot to explore the relationship between three variables. These plots display two independent variables (extrusion wheel velocity, feedstock temperature) and one dependent variable (effective stresses). In addition, the plot shows values of the effective stresses variable for combinations of the extrusion wheel velocity and feedstock temperature variables, it indicates that the lowest effective stresses during the processes occur near (8-12) rpm extrusion wheel velocity and 600 °C feedstock temperature, resulting in the optimum load required for the deformation of feedstock material during continuous forming processes.

**Fig. 8:**
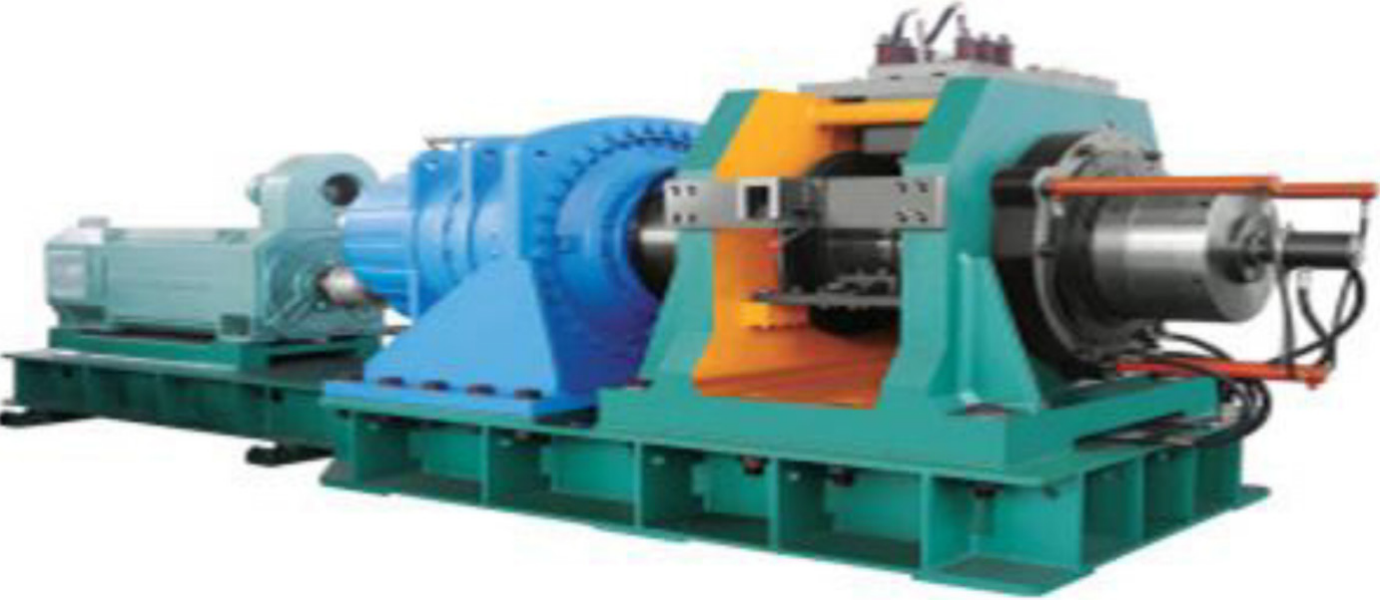
Contour plot of effective stress vs wheel velocity and feedstock temperature.

#### 2.3.2 Grey relational analysis

Grey relational analysis is the multiple response optimization techniques used for solving interrelationships among the multiple responses. A grey relational grade is obtained to analyze the multiple responses’ relational degree in this approach. [6] Have attempted a grey relational-based approach to solve multi-response problems in the Taguchi methods. The original response data was Transformed into an S/N ratio (*η*) using the appropriate formulae depending on the type of quality characteristic. Eqn. 6 expresses the smaller-the better quality characteristics. The S/N ratio (η) is given by:

(14) $$\eta =\;-10\;\text{log}\;\left[\;\frac{1}{n}\;\Sigma \;{\left(Yij\right)}^{2}\right]$$

Where n- is the number of replications

Normalization is a transformation performed on a single input to distribute the data evenly and scale it into an acceptable range for further analysis. Normalize *Y_ij_* as *Z_ij_* (0 ≤ *Z_ij_* ≤1) by the following formula to avoid the effect of using different units and to reduce variability,

(15) $$Z_{ij}\;=\;\frac{maxYij-\;Yij}{maxYij-minYij\;}$$

Grey relational coefficient is used for determining how close *Y_oj_* and *Y_ij._* Grey relational coefficient can be calculated by using the following relations,

(16) $$GC_{ij\;}=\;\frac{\Delta \min+\;\lambda \Delta max}{\Delta ij+\;\lambda \Delta max}$$

Where i = 1, 2… n - experiments and j = 1, 2...., m – responses

*GCij* = grey relational coefficient for the *i* th experiment/trial and *j* th dependent variable response
Δ= absolute difference between *Y_oj_* and *Y_ij_* which is a deviation from target the value and can be treated as a quality loss.
*Y_oj_* = optimum performance value or the ideal normalized value of the *j*_th_ response
*Y_ij_* = the *i*_th_ normalized value of the *j*_th_ response/dependent variable
Δmin = minimum value of Δ
Δmax = maximum value of Δ
λ*-* is the distinguishing coefficient which is defined in the range 0 ≤ λ≤ 1 (the value may be adjusted on the practical needs of the system)

Grey relational grade determines the optimum points of multi-response by calculating the average values of grey relational coefficient of responses at each experiment trial. So, the optimum value of each response is considered at the highest value of grey relational grade. The following relation can determine Grey's relational grade,

(17) $$G_{i}\;=\;\frac{1}{m}\Sigma GC_{ij}$$

Where GC_ij_ is Grey relational grade and *m* is the number of responses.

## Funding

No funding has been done for this research work.

## CRediT authorship contribution statement

**Mulualem Hailu Besha:** Investigation, Writing – original draft. **Devendra Kumar Sinha:** Writing – review & editing, Supervision. **Getenet Asrat:** . **Dawit Gudeta:** .

## Declaration of Competing Interest

The authors declare that they have no competing interest.

## Data Availability

Dataset for: Deformation behaviour of CP-titanium grade 2 material through continuous extrusion processes (Original data) (Mendeley Data).
